# The Structure of Digestive Tract Coordinating Digestion and Respiration in an Air-Breathing Weatherloach, *Misgurnus anguillicaudatus*

**DOI:** 10.3390/biology13060381

**Published:** 2024-05-25

**Authors:** Zixin Qi, Hongbo Ma, Li Ma, Xuefen Yang

**Affiliations:** 1 College of Animal Science and Technology, Henan University of Animal Husbandry and Economy, Zhengzhou 450046, China; qizixin2000@sina.com; 2 College of Fisheries, Huazhong Agricultural University, Wuhan 430070, China; 13175089071@163.com (H.M.); mali19980308@163.com (L.M.)

**Keywords:** air-breathing, digestive tract, histology, barium meal, evacuation rate, *Misgurnus anguillicaudatus*

## Abstract

**Simple Summary:**

Fish digestive tracts always show remarkable morphological differences depending on their functions. The weatherloach, *Misgurnus anguillicaudatus*, is a facultative air-breathing teleost fish, which employs its partial digestive tract as an accessory respiratory organ. The specificity of its digestive tract structure and function is unclear. To clarify how the digestive tract of the weatherloach serves a dual function of digestion and respiration simultaneously, the histological structures of its digestive tract, the passage of digesta and air passing through its intestine and the rate of intestinal evacuation have been studied. The study also explored the structural features of the different regions of the weatherloach intestine and how these structures coordinate digestion and respiration. This provides basic data for the coordination between digestion and respiration in the weatherloach. The results showed that the weatherloach intestine is mainly divided into five functional regions with specific structures. The process of gastrointestinal air breathing can not only aid respiration, but also promotes the excretion of the contents of the intestine. This study can support further exploration of the physiological mechanisms of gastrointestinal air breathing in fish.

**Abstract:**

To clarify how the digestive tract of the weatherloach, *Misgurnus anguillicaudatus*, serves a dual function of digestion and respiration simultaneously, the histological structures of its digestive tract, the passage of digesta and air passing through its intestine and the rate of intestinal evacuation have been studied. The results indicate that the digestive tract is divided into five functional regions, i.e., esophagus, anterior intestine, middle intestine, posterior intestine and rectum. The diverse intestinal structures have the specialized function of coordinating digestion and respiration. An X-ray barium meal examination showed in the normal breathing state, the contents of the intestine are diffusely semifluid, and air is distributed as bubbles in the dorsal intestine 2 h after feeding. After 5 h, the contents accumulated in the mid and posterior intestine, and gas flowed above the contents as bundles. After 8 h, the intestinal food was basically evacuated. In the intestinal air-breathing restricted group, the contents of the intestine remained diffuse, and a large number of digesta entered and remained in the rectum after 5 h. After the inhibition was relieved, the contents of the rectum were rapidly discharged. Measurement of the intestinal evacuation rate in the intestine showed that the evacuation of the intestinal contents lagged behind that of the normal group in the air-breathing restricted group. Compared to the normal state and inhibited GAB (gastrointestinal air breathing), we could deduce that GAB could promote the movement of the intestine.

## 1. Introduction

Fish digestive tracts always show remarkable morphological differences. Although the digestive tract appears to be a unified organ, it has been divided into a collection of related segments based on their function, location and internal anatomy. All these segments possess different functional attributes and team up to provide the functionality of the whole gut [1]. 

The weatherloach, *M. anguillicaudatus*, is a facultative air-breathing teleost fish, which employs its partial digestive tract as an accessory respiratory organ. The digestive tract is distinguished by a relatively short length and small volume. The ratio of intestinal length to body length is notably low, which is considerably below that observed in fish belonging to the Callichthyidae, which also exhibit intestinal breathing [1,2]. During gastrointestinal air breathing (GAB), they need to inhale through the mouth, then transport the gas to the posterior intestine for gas exchange and finally expel the exhaust gas through the anus [3]. Almost all studies of *M. anguillicaudatus* have focused on the respiratory region and physiological aspects of gas exchange. The posterior intestine in *M. anguillicaudatus* is highly modified, being thin-walled, air-filled and highly vascularized [4]. These modifications make it unsuitable for digestive functions, and it is generally found to be devoid of digesta in this region. The digesta can rapidly pass the respiratory region under the pressure from the anterior regions [5], and at times the air is excreted under contraction. But how this is done is not clear since the anterior intestine is always full of food that would seriously hamper the passage of airflow. Zhang et al. proposed that it is the disorder between the digestion and breathing that leads to a high mortality in the aquaculture [6].

It is likely that the contractions of muscles in the middle spiral rotation compress digesta into a narrow string before entering the posterior respiratory region, according to McMahon’s research [3]. Actually, the mucosa and muscle layer in the spiral region are very undeveloped [7]. Additionally, there exists a phenomenon that the digesta in this region are coated gradually by a layer of the mucus sac [5]. Little is known about the specific structures and functions of other regions.

A barium meal is a diagnostic procedure which uses X-ray imaging to detect the abnormalities in the esophagus, stomach and small intestine [8]. The gastrointestinal tract, like other soft tissue structures, does not show up clearly enough on plain radiographs for diagnostic purposes. The barium salt is radiopaque and easily distinguishable on radiographs [9]. The barium meal experiment is capable of providing a visual representation of the intestinal structure and the relative positioning of the contents and gas, which is an invaluable tool for investigating intestinal peristalsis in the weatherloach.

In contrast to some other GAB fish (Callichthyidae), *M. anguillicaudatus* is a gastric fish and has a simpler and shorter gut. The GAB process of the weatherloach has been described [3]. It was found that there may be a mechanism for coordination between digestion and respiration. In order to clarify the coordination between digestion and air breathing, this study was conducted to investigate the digesta and ventilation following barium meal ingestion in conjunction with X-ray examination, determine the rate of intestinal evacuation and also examine the histological structures of the intestinal tract using light microscopy (LM) and scanning electron microscopy (SEM). The findings contribute to the establishment of a theoretical framework for the healthy breathing process of the weatherloach.

## 2. Materials and Methods

### 2.1. Animals

Two hundred adult weatherloaches, *M. anguillicaudatus* (the average body weight and body length were 14.22 ± 4.27 g and 11.74 ± 1.60 cm, respectively), were bought from the Baishazhou Aquatic Products Market and raised in the Aquaculture Base of the College of Fisheries, Huazhong Agricultural University in Wuhan, Hubei Province, China. All the fish were acclimated at 20 °C~26 °C and DO > 6 mg/L prior to experimental use. After 2 weeks of temporary rearing to acclimatize the fish to the experimental conditions, all the fish being investigated were starved for 24 h. All the experimental procedures involving fish were approved by the institution’s animal care and use committee of the Huazhong Agricultural University, China.

### 2.2. Histology 

Ten adult fish (five for LM and five for SEM) were euthanized using tricainemethanesulfonate (MS-222, 100 mg L^−1^). After dissecting them, the specimens’ intestinal tracts were picked out and divided into smaller samples (0.5–1.0 mm) based on the external morphology. The samples for LM were fixed in Bouin’s fluid, dehydrated in a series of alcohols (70%, 80%, 90%, 95% and 100%), and finally embedded in paraffin blocks. The whole digestive tract was sectioned at 5–7 μm and stained with hematoxylin and eosin. Histological sections were observed and photographed via microscopy (Axio Imager A2, Zeiss, Jena, Germany). For SEM analysis, the samples were cut into an appropriate size (2 × 2 mm^2^), fixed in glutaraldehyde and dehydrated, dried in a critical point drier, mounted on aluminum stubs, vacuum coated with gold and then examined via SEM (JSM-6390 LV, Tokyo, Japan) operating between 10 and 30 kV.

### 2.3. X-ray Study of the Intestinal Contents

A pre-experiment showed that the proper addition of barium sulfate (BaSO_4_) to feed had little effect on the appetite of *M. anguillicaudatus*, and the digesta took 7–8 h to pass through the GI tract. The experiments were conducted at 20 °C~26 °C and in a DO > 6 mg/L environment. Based on this result, thirty loaches in the present study were divided into two groups (the normal group and intestinal air-breathing restricted group) and cultured in the same plastic tank, which was divided into two parts using mesh. The intestinal air-breathing restricted group was covered by mesh under water to prevent air breathing at the surface. The fish were fed with excess barium meal (eel powder feed and BaSO_4_ were mixed in the ratio of 2 to 1). The remaining feed was cleaned up half an hour after feeding. In the control group, three fish were randomly sampled at 0 h, 2 h, 5 h, 8 h post-feeding. In the restricted group, the first sampling was at 8 h post-feeding. After that, the mesh was removed, and the fish came to the surface to take their first breath of air. The second and third samplings were performed after the first air breath and half an hour later, respectively. The sampled fish were narcotized and photographed under an X-ray generator. The operating parameters of the X-ray generator were 40.00 KVP, 12.50 ms and 2.50 mAs (the optimal parameter setting, balancing the lowest radiation dose with the highest imaging quality, was achieved through rigorous verification processes).

### 2.4. Measurement of the Intestinal Evacuation Rate

The remaining fish were divided into two groups under the same conditions. Ten fish (five in each group) were randomly sampled every hour from 1 to 7 h after feeding. The fish were weighed and killed under anesthesia. The contents were extracted from the bulbous section (called the digestive intestine), intermediate spiral section (called the spiral intestine) and rectum section of the intestine (called the respiratory intestine) and weighed. 

### 2.5. Statistical Analysis

Evaluations of the mucous cell and erythrocyte were made using LM on randomly selected 100 μm lengths of mucosal epithelium from the consecutive five whole-mount preparations [10]. The relative blood capillary density was reflected by the number of erythrocytes. The digestive tract was composed of four layers: mucosa, submucosa, muscularis and serosa. The general four layers were measured using WCIF ImageJ (Win 64-bit). The proportion of the contents of each intestinal segment to the net weight was also calculated. Excel 2016 and SPSS 19.0 software packages were used to perform the statistical analysis. All the statistics were analyzed via one-way analysis of variance and represented by the mean ± standard deviation. The different letters in the same column show the significant difference at the 5% level in the tables.

## 3. Results

### 3.1. Morphological and Histological Observation

**Figure 1:**
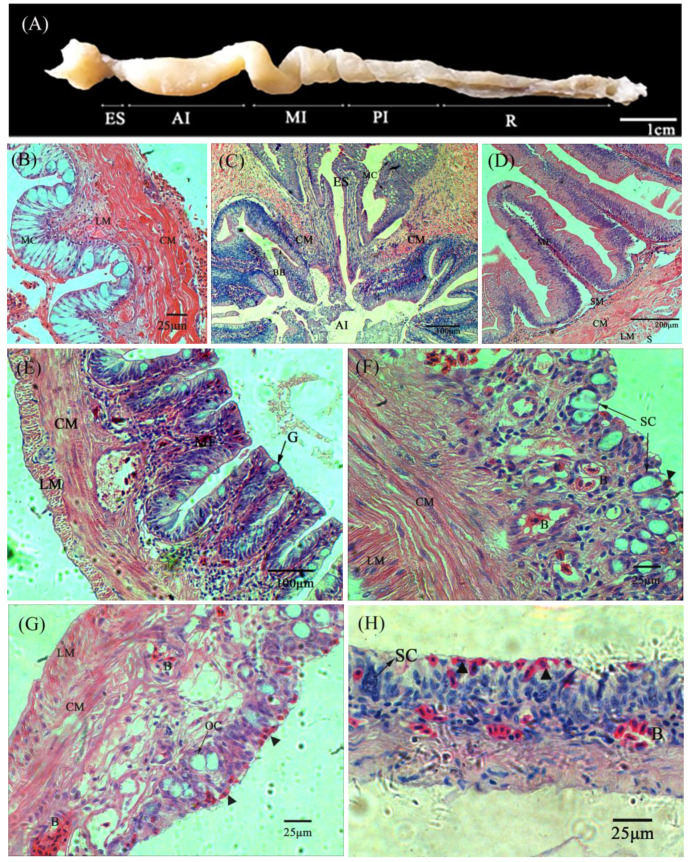
General and microanatomy of the digestive tract of *M. anguillicaudatus*. (**A**), Schematic view of the laid-out excised digestive tract. (**B**), 400×, Transverse section of the esophagus with abundant large saccular mucous cells. (**C**), 100×, Longitudinal section through the esogaster-intestinal juncture showing the funnel-like valve. (**D**), 40× Transverse section of the anterior intestine with mucosal fold. (**E**), 100×, Transverse section of the middle intestine with flattened mucosal folds. (**F**), 400×, Transverse section of the ventral of posterior intestine. There are numerous large saccular mucous cells around slight red blood cells. (**G**), 400×, Transverse section of the dorsal of posterior intestine. The oval mucous cells are below and visible red blood cells are present. (**H**), 400×, Transverse section of the rectum. Blood cells are distributed near the outermost epithelial layer. Abbreviations: AI, anterior intestine; B, blood vessels; BB, brush border; CM, circular muscle layer; ES, esophagus; G, goblet cell; LM, longitudinal muscle layer; MF, mucosal fold; MI, middle intestine; OC, oval mucous cells; PI posterior intestine; R, rectum; S, serosa; SC, saccular mucous cells; SM, submucosa; ▲, erythrocyte. Histological sections were stained with hematoxylin and eosin (**A**–**H**). Scale bar is shown in the figure.

The digestive tract of *M. anguillicaudatus* is a short and straight tube (Figure 1). The ratio of the length of the gut to the body length was 0.52. From a gross anatomical view, the anterior inflated portion is separated by an intermediate spiral section from the posterior respiratory zone. The histological studies allowed a more precise identification of the digestive tract into five different regions, namely esophagus, anterior intestine, middle intestine, posterior intestine and rectum. All these regions are composed of four layers: mucosa, submucosa, muscularis and serosa. The layers in the different regions of the digestive tract showed significant differences (Table 1). 

#### 3.1.1. Esophagus

The esophagus is the narrowest area in the digestive tract. The observation of transverse sections showed that the mucosa is covered by a stratified squamous epithelium thrown into 12–14 longitudinal folds. Numerous mucous cells are tightly lined among the epithelium (Figure 1B). The muscular layer is composed of the inner longitudinal and outer circular striated muscle fibers. At the junction with the intestine, the muscularis becomes thicker and forms an inner funnel-like valve with a strong circular muscle stretching into the intestine (Figure 1C). After this valve, the stratified squamous epithelium of the esophagus makes an abrupt transition to the simple columnar epithelium of the intestinal mucosa.

#### 3.1.2. Anterior Intestine

**Figure 2:**
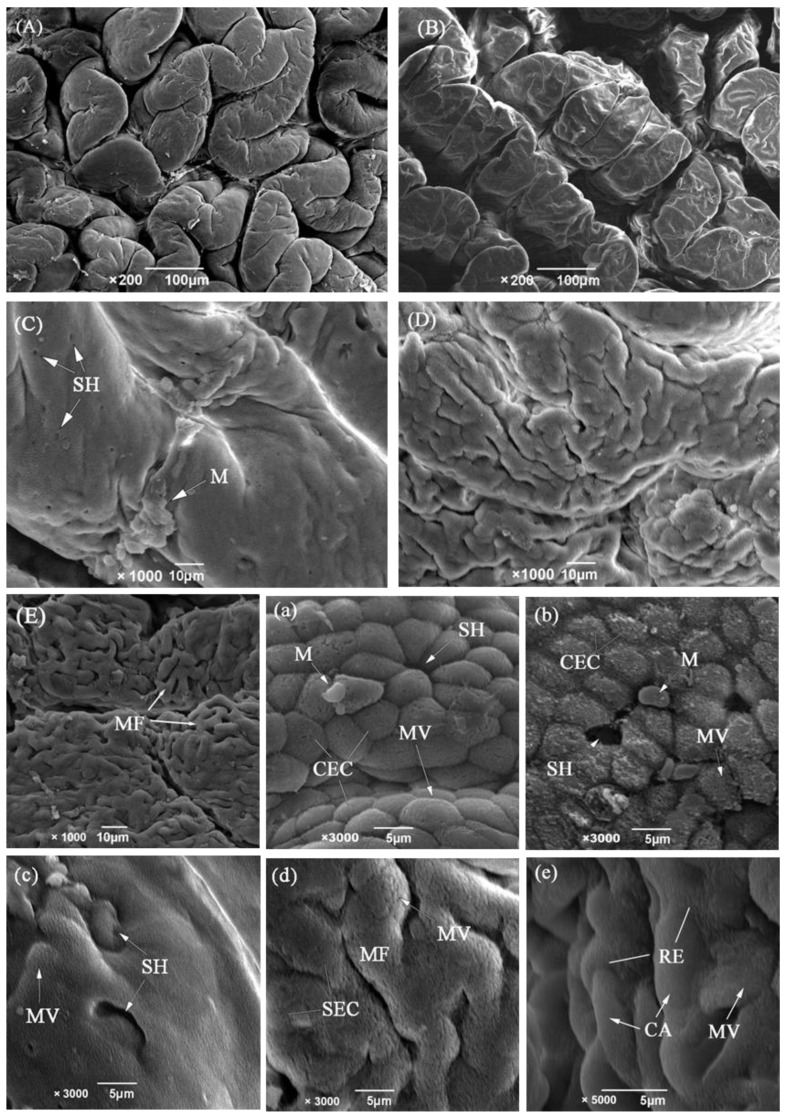
SEM of the different regions of the intestine of *M. anguillicaudatus*. (**A**) Irregular mucosal folds in the anterior intestine. (**B**) Longitudinal zigzag pattern and flattened folds in the middle intestine. (**C**) Luminal surface of the ventral of posterior intestine showing massive mucus and secretion pits. (**D**) Rough surface of the dorsal of posterior intestine showing microfolds (MF) on the primary folds. (**E**) Microfolds in the rectum showing massive blood capillaries. (**a**–**e**) are corresponding higher-power amplification; note the presence of microvilli (MV) on the surface of the columnar epithelial cells.

The anterior intestine was found to be bulged to form an intestinal bulb of 3.26 mm in diameter. The wall was strongly convoluted into 35–43 longitudinal mucosal folds (Figure 2A). The mucosal folds were typically finger-shaped, 459.26 ± 100.68 μm in height and equivalent to 1/4 of the interior intestine diameter. Under SEM, the folds were irregular, and the columnar epithelial cells were tall and cylindrical with dense microvilli (Figure 2a). Goblet cells were scattered among the epithelial cells. The submucosa was very thin and indistinguishable from the mucosa (Figure 1D). The muscularis was the thickest and consisted of inner circular muscle (148.08 ± 17.29 μm) and outer longitudinal muscle layers (86.92 ± 11.90 μm). At the terminal of the intestinal bulb, the intestine tended to narrow (width: 1.8–2.1 mm) and curved backwards strongly just like a pylorus in the stomach. 

#### 3.1.3. Middle Intestine

The middle intestine, in comparison to the anterior intestine, is a narrow and slightly spiral tube. It is also strongly muscular and the thickness of the inner circular muscle is about twice as thick as that of the outer longitudinal muscle. The mucosal folds become lower (197.58 ± 20.32 μm) and less numerous (28–35). Furthermore the shape of folds changes to be trapeziform or square distortions (Figure 1E). SEM showed that the mucosal folds are in a zigzag pattern, and the surface is flattened out to such an extent that it resembles pavement (Figure 2B). The brush border on the intestine’s epithelial cells also decline somewhat in height according to SEM (Figure 2b). 

#### 3.1.4. Posterior Intestine

The posterior intestine is translucent, spiral and strongly twisted into some sacculated structures, which result in an increased lumen volume. The mucosal folds were no longer obvious, but the submucosa was markedly increased with collagen fibers (Table 1, Figure 1G). The columnar epithelium was replaced by squamous cells and contained massive mucous cells and capillaries. The number of blood capillaries invading the dorsal intestinal lumen was more significant than that in the ventral lumen (*p* < 0.05) (Figure 1F, Table 2). The SEM study showed that the mucosal surface is rough with abundant microfolds on the dorsal mucosal folds (Figure 2D,d), while it is relatively smooth with a great number of secretion pits on the ventral side (Figure 2C,c). Mucous cells on the ventral side are increased in number and size when compared with the dorsal zones. In addition, the mucous cells in the ventral mucosa are always sacciform and opening to the surface. However, the mucous cells on the dorsal side are globular and still distant from the gut lumen.

#### 3.1.5. Rectum

The rectum contains all of the histological elements of the preceding sections. But the layers are significantly thinner. The mucous cells with an ovoid or spherical shape are present, but fewer than that in the other sections (Table 2). The epithelial layer of the rectum contains many more blood cells and capillaries than those of the intestine (Figure 1H). Under SEM, the folds are not present and are replaced with a smooth flat surface (Figure 2E,e). The capillary network is rich and almost covers the entire surface.

### 3.2. The Digesta and Ventilation in the Intestine

**Figure 3:**
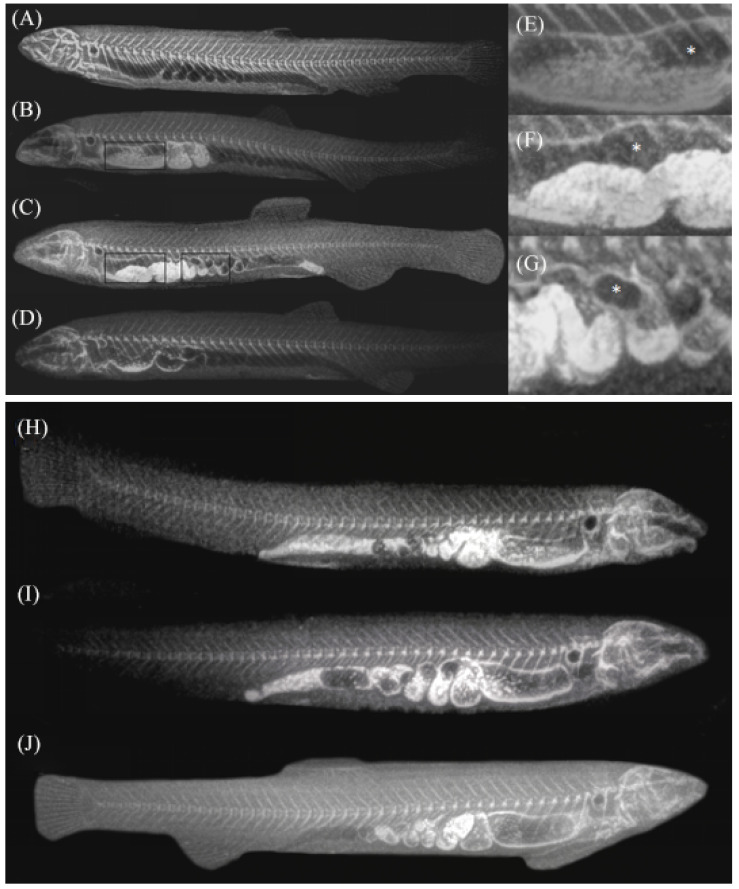
Schematic view of the digesta and ventilation in the intestine after feeding. The roentgenograms (**A**–**D**) in this figure show 0, 2, 5, 8 h post-feeding, respectively; (**E**) shows the greater multiple of the black box in (**B**); (**F**,**G**) show the greater multiple of the black boxes in (**C**); (**H**) shows eight-hour suppression of intestinal breathing; (**I**) shows the first time of air breathing after eight-hour suppression; (**J**) shows one hour after lifting of restrictions of eight-hour suppression of GAB; * represents gas and the white strips represent food in the intestine.

In the roentgenogram, the black area in the intestine represents the existence of gas. It is clear that eating a barium meal could cause the intestine to turn white. Under the normal condition, the digesta take 7 h~8 h to pass through the alimentary intestine. The digesta were mainly huddled in the anterior intestine within the 2 h post-feeding. And it was propelled into the spiral intestine in the 5 h post-feeding, then soon excreted positively (Figure 3B,C). The food was ropy and semi-liquid in the anterior intestine, gradually turning into some strings of slightly compressed strips in the middle intestine before excretion. These strips clung to the ventral lumen of the posterior intestine, leaving an air channel in the dorsal part. However, it was rarely seen that the digesta stayed in the rectum from feeding to expulsion. There was always a gas but not with a constant volume in the anterior intestine even though *M. anguillicaudatus* was feeding. The swallowed air was thrusted past digesta into the dorsal side of the anterior intestine and formed one to two large bubbles above the digesta (Figure 3E). The ventilation was smoother in the latter parts of the digestive tract because the irregular spirals were in the form of air sacs with the digesta sticking to the bottom and air above which created an air channel (Figure 3G). 

*M. anguillicaudatus* that were prevented from performing air breathing were observed to be swimming against the surface barrier after feeding. However, there were still a lot of digesta in the spiral intestine and not as strips compared to the fish that were allowed to perform air breathing 8 h after feeding. There was much spillover of digesta from the spiral intestine into the rectum (Figure 3H) 4 h after feeding which was not discharged. Once the restrictions were removed, the gas filled the rectum and expelled the digesta from the body quickly (Figure 3I,J).

### 3.3. Characteristics of Intestinal Evacuation

**Figure 4:**
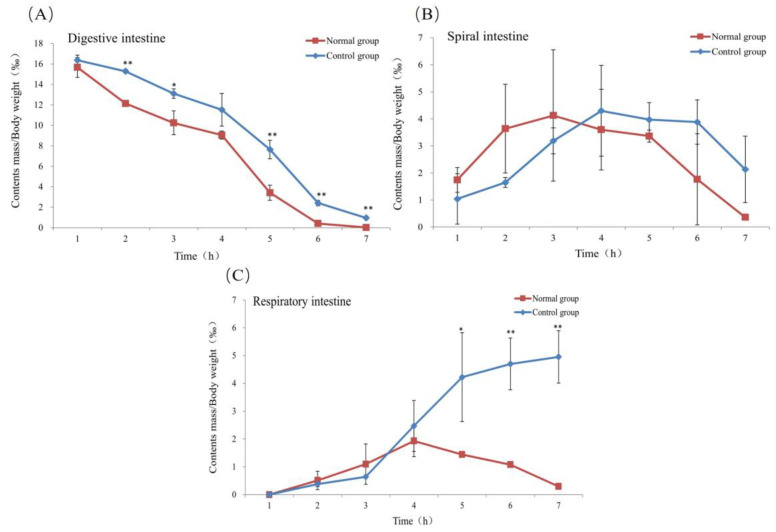
Changes in contents in different sections of the intestinal tract. (**A**), Digestive intestine; (**B**), Spiral intestine; (**C**), Respiratory intestine. *, represents significant differences from control group at the same time (*p* < 0.05); **, represents extremely significant differences from control group at the same time (*p* < 0.01).

The contents as a proportion of body weight in the digestive intestine showed a gradual decrease in both groups over time, from 15.59‰ to 0.02‰ in the normal group and from 16.39‰ to 0.96‰ in the control group at 7 h (Figure 4A). It can be seen that the differences were significant (*p* < 0.05) or highly significant (*p* < 0.01) except at 1 h and 4 h when the differences were not significant. The trend of the curves showed that the control group significantly lagged behind the normal group.

The contents of the spiral intestine of both groups showed the same trend of increasing and then decreasing at 7 h. The normal group’s ratio decreased from 1.78‰ to 0.36‰, whereas the control group’s decreased from 1.09‰ to 2.12‰ (Figure 4B). At 7 h, the control group’s contents ratio remained much higher than that of the normal group, and the difference between the two groups was not significant. The change in contents over time in the spiral intestine control group lagged behind that of the normal group. 

The respiratory intestines of both groups were almost devoid of contents at 1 h, and both groups showed a slow rising trend before 4 h (Figure 4C). The control group showed an increasing trend, reaching a maximum value of 4.93‰ at 7 h. But the normal group showed a decreasing inflection point after 4 h, with only a small amount of residue remaining at 7 h. The performance of the two groups appeared different after 4 h.

## 4. Discussion

### 4.1. Intestinal Modifications for Different Functions

The prevailing view is to divide the tract of *M. anguillicaudatus* into three functional regions: the foregut, midgut and hindgut, respectively, corresponding to the digestive, transitional and respiratory regions [3,4]. From the present results of the structural observations, the intestine of *M. anguillicaudatus* appears to possess five functional regions. In the esophagus, the mucosal epithelium is stratified and dominated by large saccular mucus-secreting cells at its surface. Park et al. have described that these mucous cells could increase the lubrication action during the transportation of food [10]. An esophageal-intestinal valve lies directly behind the esophagus. Generally, most agastric fish have a short and muscular esophagus, which does not require a posterior closing apparatus, demonstrating that the esogaster-intestinal valve is functionally related to the pyloric sphincter of gastric fish [11,12,13]. Therefore, in functional terms, the presence of the esogaster-intestinal valve in *M. anguillicaudatus* is presumed to perform as a one-way valve, allowing food and gas to pass through into the intestine and effectively prevent the reverse flow.

The anterior intestine is bulged to form a pseudogaster. Under SEM, the luminal surface of the anterior intestine is lined with prominent columnar epithelial cells with dense microvilli, indicating that the anterior intestine performs the major functions of digestion and absorption. The heavy folds, thin lamina propria and thick muscular walls have been considered to be the results of expansion, and the satiation-induced intestinal distension threshold in fish has not yet been reached [14]. Kariya et al. suggested that gas is more likely to cause gastrointestinal dilatation in fish than solid or liquid food [15]. In the roentgenogram, the anterior intestine was significantly enlarged 2–5 h post-feeding, and gas was present above the food in the form of two to three large bubbles. Therefore, the anterior intestine also functions as temporary gas storage although it is filled with food. 

The middle intestine is also strongly muscular as in the anterior intestine, but the columnar epithelium of the middle intestine is reduced. The microvilli become sparse and decreased in height. The mucosal folds become gradually flattened out and transform into a trapeziform distortion. All these geometric distortions, also called flattened folds, have a flat top and together form a smooth inner surface [13]. Some similar structures have been occasionally observed in the bends of the digestive tract, spiral valves or intestinal-rectal junction which are always designed to slow the movement of food [16,17]. The cause of these geometric distortions is often the extrusion of the food. Combining these geometric distortions with the features of a narrow diameter and abundant striated muscle [18] in this region, we can speculate that the middle intestine provides an added function of compressing food boluses into bars in *M. anguillicaudatus*.

The epithelium is flattened in the posterior intestine and more like the respiratory epithelia [19]. The remarkable features in the posterior intestine are the existence of numerous mucous cells and capillaries within the mucosal epithelium. The histological difference between the dorsal and ventral intestinal lumen suggests the different functions. In this region, the ventral intestinal lumen is always in contact with the digesta, but the dorsal lumen is usually exposed to air. The smooth surface with numerous mucous cells opening to the ventral lumen and expansion with air could contribute to secreting mucus to transport the digesta [20]. Our observations also verified that a thick mucus layer is present on the lumenal surface and provides explicit morphological and histological evidence of the so-called functional region [21] as the formation of the mucus sac surrounding the fecal material. The present research also showed that there are more capillaries in the dorsal part than in the ventral part. The capillaries are just below the flattened epithelium and have short air–blood diffusion distances (<2 μm), which means that the dorsal part of the posterior intestine is suitable for gas exchange. In addition, the dorsal epithelium also presents with slightly elevated respiratory islets with covering flattened cells [22]. Microfolds have been observed exclusively on the respiratory surface, such as the esophagus in *Dallia pectoralis* [23] and stomach in *Pterygoplichthys anisitsi* [24] and, actually, are essentially caused by the bulging of blood capillaries [25,26]. Thus, the appearance of the capillaries in the dorsal mucosa indicates its adaptation to the long-term exposure to air in the posterior intestine.

A typical respiratory epithelium can be seen in the rectum. The epithelium is completely flattened and thinner so as to reduce the diffusion distance across the luminal boundary. The existence of surfactant-producing lamellar bodies in the respiratory intestinal epithelia has been confirmed in *Misgurnus* [27]. The microvilli are still observed, although they have been seriously degraded. Gonçalves et al. and Huang et al. suggested that the respiratory intestinal segment still has the function of nutrient uptake [4,28]. However, this region seldom has the time to uptake nutrients because it is generally devoid of digesta except for some unusual cases such as restricting the air breathing in our experiment. The very small number of goblet cells in the rectum of *M. anguillicaudatus* makes this species an exception among teleosts. The advantages of mucus may include lubrication for the passage of digesta and the protection of the thin-walled, highly vascularized posterior intestine from desiccation and mechanical damage, but excessive mucus may increase the gas diffusional distance.

### 4.2. Coordination between Digestion and Respiration

This study supports the suggestion by Kramer that the intestine of *Corydoras aeneus* allows simultaneous processes of digestion and respiration [29]. In *M. anguillicaudatus*, there exists a mechanism of coordinating the transport of air with the transport of digesta. X-ray examination indicated that the anterior intestine always reserves several bubbles although it is filled with digesta. It is hypothesized that these bubbles could act as a backup gas channel for ventilation to reduce the risk of dead space when a fish is forcing air down into the posterior segments. Similar to the anterior digestive intestine of GAB fish *Holposternum littorale* and *C. aeneus* that is described by Persaud et al. [5], the middle intestine of *M. anguillicaudatus* also plays an important role in initially processing the digesta. The compacted digesta would be then wrapped with mucus. The packaging of digesta in the form of a mucous bar not only creates an air channel above the digesta and ensures that the majority of the dorsal epithelium is in contact with air, but also makes transportation quicker in the rectum.

Since the rectum is modified into a thin-walled structure, it is not enough to transport feces out of the body. Most authors describe the ventilation in the intestine as being responsible for the passage of digesta through the respiratory intestine, but have never verified this [3,5,30]. Our study shows that the intestinal contents of the control group were rapidly excreted by the propulsion of intestinal gas, while the intestinal air-breathing restricted group had a more delayed rate of intestinal contents emptying due to the lack of propulsion, and a large amount of food debris accumulated in the rectum (Figure 4). Boujard et al. reported that food intake and air breathing increased simultaneously and air breathing in fasted *H. littorale* is always lower than that in feeding *H. littorale* [31]. These fish are therefore air breathing for some reason other than meeting respiratory requirements. Our result proves that one important purpose of GAB is to transport feces through the rectum quickly and not only to meet oxygen requirements. This mechanism ensures that the rectum is clear of feces and can function as an efficient gas exchange organ without disrupting the gas exchange process for long periods. 

## 5. Conclusions

The present investigation provides morphological, histological and intestinal evacuation rates evidencing that digestion is coordinated with GAB. The anterior intestine is the main digestion and absorption area. The middle intestine has the added function of compressing food so as to create an air channel in the posterior intestine. The posterior intestine can secrete mucus to wrap the feces to aid defecation via the rectum. Weatherloaches rely on a specific intestinal structure to coordinate digestion and respiration at the same time. In addition to its role in assisting respiration, GAB not only accelerates the transportation of the digesta in the digestive and spiral intestine, but it is also essential to move digesta through the poorly muscled respiratory intestine. 

## Figures and Tables

**Table 1:** Features of tissue in the digestive tract of *M. anguillicaudatus* (Mean ± deviation).

	Mucosal Folds Height (μm)	Submucosa Thick (μm)	Circular Muscle Thick (μm)	Longitudinal Muscle Thick (μm)	Serosa Thick (μm)
Esophagus	94.32 ± 18.87 ^a^	14.22 ± 5.43 ^a^	36.65 ± 5.88 ^c^	18.64 ± 7.99 ^b^	4.89 ± 1.00 ^a^
Anterior intestine	459.26 ± 100.68 ^b^	29.98 ± 7.49 ^b^	148.08 ± 17.29 ^e^	86.92 ± 11.90 ^c^	5.22 ± 1.01 ^a^
Middle intestine	197.58 ± 20.32 ^c^	24.96 ± 5.74 ^b^	88.52 ± 12.61 ^d^	35.94 ± 5.87 ^d^	4.39 ± 1.12 ^a^
Posterior intestine (Dorsal)	-	53.83 ± 8.34 ^c^	17.23 ± 3.16 ^a^	31.68 ± 4.5 ^d^	4.23 ± 1.23 ^a^
Posterior intestine (Ventral)	-	24.98 ± 5.49 ^b^	24.83 ± 3.34 ^b^	19.32 ± 4.18 ^b^	4.96 ± 0.99 ^a^
Rectum	-	12.23 ± 3.21 ^a^	20.42 ± 3.41 ^ab^	6.90 ± 1.71 ^a^	4.23 ± 0.82 ^a^

Note: The letters (a–e) indicate that those marked with the same letter indicate that the difference between the groups is not significant (*p* > 0.05), whereas those with different letters indicate that the difference between the groups is significant (*p* < 0.05). The same is true below.

**Table 2:** The linear density of erythrocytes (blood capillaries) and mucous cells in the mucosal epithelium of the digestive tract in *M. anguillicaudatus* (Mean ± deviation).

Digestive Tract	No. of Mucous Cells per 100 μm	No. of Erythrocyte per 100 μm (Blood Capillaries)
Esophagus	25.1 ± 3.16 ^e^	-
Anterior intestine	9.6 ± 1.85 ^c^	-
Middle intestine	4.8 ± 1.17 ^b^	2.6 ± 0.38 ^a^ (+)
Posterior intestine (Dorsal)	16.0 ± 1.72 ^d^	10.2 ± 1.40 ^b^ (+++)
Posterior intestine (Ventral)	12.3 ± 1.50 ^c^	3.9 ± 0.60 ^a^ (++)
Rectum	2.3 ± 0.8 ^a^	17.3 ± 1.88 ^c^ (++++)

Note: +, ++, +++, ++++, gradual increasing relative capillary density; -, absent.

## Data Availability

The data presented in this study are available on request from the corresponding author.

## Abbreviations

The following abbreviations are used in this manuscript:

GAB	Gastrointestinal Air Breathing
LM	Light Microscopy
SEM	Scanning Electron Microscopy
GI	Gastrointestinal
ES	Esophagus
AI	Anterior Intestine
MI	Middle Intestine
PI	Posterior Intestine
R	Rectum
